# The Dynamics of Glutathione Species and Ophthalmate Concentrations in Plasma from the VX2 Rabbit Model of Secondary Liver Tumors

**DOI:** 10.1155/2011/709052

**Published:** 2011-02-17

**Authors:** R. Abbas, R. S. Kombu, R. A. Ibarra, K. K. Goyal, H. Brunengraber, J. R. Sanabria

**Affiliations:** ^1^Division of Transplant and Hepatobiliary Surgery, Department of Surgery, Case Western Reserve University School of Medicine and University Hospitals, Case Medical Center, 11100 Euclid Avenue, Lakeside 7510, PS 5047, Cleveland, OH 44106, USA; ^2^Department of Nutrition, Case Western Reserve University School of Medicine and University Hospitals, Case Medical Center, 11100 Euclid Avenue, Lakeside 7510, PS 5047, Cleveland, OH 44106, USA

## Abstract

*Purpose*. Available tumor markers have low sensitivity/specificity for the diagnosis of liver tumors. The present study was designed to evaluate the oxidoreductive status of the liver as surrogates of tumor subsistence and growth. *Methods*. Glutathione species (GSH:GSSG), ophthalmate (OA) concentrations, and their turnover were measured in plasma of rabbits (*n* = 6) in their healthy state and in the state of tumor growth after implantation of the VX2 carcinoma in their liver. Tumors were allowed to grow for a period of 14 days when rabbits were sacrificed. Livers were removed and cysteine concentration was measured in liver tissue. *Results*. Tumor growth was found in 100% of the rabbits. Concentration and labeling of GSH/GSSG were similar in experimental animals before and after tumor implantation and to sham animals. In contrast, OA concentration increased significantly in experimental animals after tumor implantation when compared to same animals prior to tumor implantation and to sham animals (*P* < .05). The concentration of cysteine, a precursor of GSH, was found to be significantly lower in the liver tissue adjacent to the tumor (*P* < .05). *Conclusion*. Disturbances in the oxidoreductive state of livers appear to be a surrogate of early tumor growth.

## 1. Introduction

Liver tumors are the third most common malignancies of the gastrointestinal tract worldwide [1]. In the Western World, secondary liver tumors are more frequent than primary ones, even though the incidence of hepatocellular carcinoma (HCC) has been increasing over the last 20 years, especially in males [2]. Nearly 80% of liver tumors are diagnosed in advanced stages precluding curative therapies [3], and tumor markers for the early detection of liver growth lack high levels of sensitivity and specificity. Currently, *α*-fetoprotein (AFP) and carbohydrate antigen 19.9 (CA19.9) are the most frequently used serum markers to detect hepatocellular carcinoma and cholangiocarcinoma, respectively [4–9]. Carcinoembryonic antigen (CEA) is frequently used for detection and followup of colorectal liver metastases [10]; however it may be increased in many other medical conditions. The ideal biomarker for early detection of liver cancer would be specific for the malignant condition and sensitive enough to detect the neoplasm at an early stage, when treatment is still possible [11]. Serum metabolites related to oxidative stress are thought to be a potential biomarker for the early detection of cancer [12, 13].

Patients with advanced malignancy are typically in a catabolic state. Tumor necrosis factor-*α*(TNF-*α*)and other cytokines have been implicated in promoting a low-energy intake with increased energy expenditure and negative energy balance, likely due to tumor growth [12]. Mantovani et al. found a significant elevation in TNF and IL-6 along with a reduced activity of the enzyme glutathione peroxidase in patients with advanced stages of various cancers (stage III or IV) [13]. Glutathione peroxidase is considered a surrogate marker for body oxidative stress and its reduction may be due to a reduction of its substrate availability, reduced glutathione [12, 13]. In addition, reduced glutathione concentrations in plasma were significantly lower in patients with advanced cancer from different organs including renal, breast, lung, and colon cancers [14–16]. The glutathione (GSH:GSSG) redox couple is one of the main cellular defenses against oxidative stress [14–17]. Failure of the glutathione redox buffer system may result in mitochondrial failure and cell apoptosis. Ophthalmate (OA) is an analogue of glutathione in which the cysteine moiety is replaced by L-2-aminobutyrate, a catabolite of threonine. OA synthesis occurs when same enzymes that catalyze the assembling of glutathione find cysteine availability low [17]. It was recently reported that the concentration of ophthalmate in rat liver and plasma increases when oxidative stress is induced by acetaminophen [17]. The liver has the highest concentration of reduced glutathione in the body, but the liver ophthalmate pool is very small (1% of the size of GSH:GSSG) since it is actively transported out of the cell [17]. We hypothesized that early stages of liver tumor growth may enhance cell oxidative stress with alterations of the redox state of the glutathione system, promoting an increase in ophthalmate concentration. 

Our laboratory developed a technique of stable isotopomer analyses for the measurement of the GSH/GSSG/OA redox system by the labeling of glutathione species and OA from a deuterated water (^2^H_2_O) load [18]. In addition, the VX2 rabbit model is a reliable model of secondary liver tumors. First developed by Shope and Hurst in 1933 [19], the VX2 carcinoma is an anaplastic squamous cell carcinoma derived from a virus-induced papilloma. It has been extensively used to induce the growth of secondary liver tumors [20–24]. Thus, to test our hypothesis we made use of both the VX2 model and of new mass spectrometric techniques to measure the concentration and labeling of the GSH/GSSG/OA redox system from deuterium-enriched body water [18].

## 2. Materials and Methods

### 2.1. VX2 Cell Line

The VX2 cell line was obtained from Dr. A. Exner Laboratory (Case Western Reserve University School of Medicine, Cleveland, OH). Tumor donor rabbit (F1; *n* = 1) was developed by intramuscular injection of the VX2 cell line in the thigh muscle (Vastus lateralis) of a New Zealand white male rabbit as previously described in [25]. After four weeks, the tumor was harvested and minced into tissue cubes of 1.0 mm^3^ and saved at −70°C in FBS with 10% DMSO until implantation. On the day of tumor implantation, grafts were thawed and washed three times in Hanks' Buffered Salt Solution (HBSS). General chemicals and reagents were obtained from Sigma-Aldrich (St Louis, MO). Deuterated water (^2^H_2_O at 99.9%, glass distilled) was procured from Isotec (Miamisburg, OH). Fetal bovine serum was obtained from Cambrex (East Rutherford, NJ).

### 2.2. Animal Model

Adult New Zealand white male rabbits (*n* = 8; Covance, Princeton, NJ) weighing 2,500–3,000 g were placed in quarantine for 15 days prior to any experiment and kept under standard conditions of day cycle, temperature, and humidity with food (Rabbit chow, NJ) and water *ad libitum*. Studies on the animals were approved by the Institutional Animal Care and Use Committee (IACUC) at Case Western Reserve University and carried out according to its guidelines.

**Figure 1:**
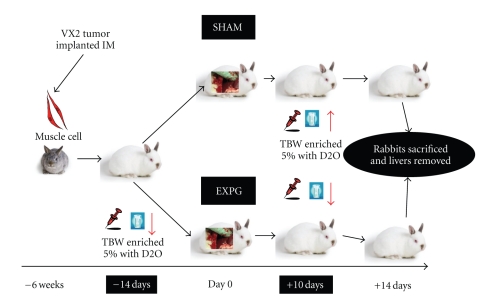
*Experimental design using the VX2 rabbit model of secondary liver tumors*. The VX2 cell line was implanted in the muscle of F1 rabbits 4 weeks prior to tumor procurement. The experimental group was formed by rabbits where 1 mm^3^ tumor grafts were implanted in their liver. The Sham group consisted of rabbits that underwent similar operation, but 1 mm^3^ pieces of gelfoam were implanted in their liver. Enrichment of body water was achieved at 5% of total body water, and blood samples were taking at days −13, −12, and −11 prior to tumor implantation and at days +11, +12, and +13 after tumor implantation.

All surgical procedures were performed under sterile conditions and under the influence of intramuscular anesthesia with Xylazine (5 mg/kg), Ketamine (50 mg/kg), and Acetylpromazine (10 mg/kg). Depth of anesthesia was monitored by recording heart rate, respiratory rate, eye reflex, and response to stimulus. Surgical site was shaved and swabbed with Providian-iodine solution (Betadine, MI) and Marcaine 0.25% without epinephrine was administered subcutaneously for local anesthesia. Tumor recipient rabbits (F2; Experimental Group, *n* = 6) underwent median laparotomy with implantation of VX2 tumors at two separate liver sites (right medial and right posterior lobes) through a 2 mm incision in the liver capsule and each site was closed and labeled with a 6 : 0 Prolene suture (Ethicon, NJ). All animals received equal tumor load. Other rabbits underwent similar operative procedures with the implantation of Gelfoam (Ethicon, NJ) as grafts. They served as a control group (F2; Sham group, *n* = 2) (Figure 1).

Rabbits were given buprenorphine (0.3 mg/mL at a dose of 0.1 mg/kg) (Sigma, MO) administered subcutaneously twice daily for two days following any procedure. Penicillin (300,000 units/mL at a dose of 50,000 units/kg) and gentamicin (40 mg/mL at a dose of 3 mg/kg) were injected subcutaneously (SC) prior to the procedure and once daily for the two postoperative days. 20 mL of normal saline (0.9% NS) was given SC after the procedure for insensible water losses. All animals were monitored three times a day for 72 hours and then twice a day until animals were sacrificed. No animals had any sign of infection or sickness.

### 2.3. Tumor Growth Assessment

Animals, two weeks after tumor implantation, underwent surgical removal of the liver. Biopsies of liver tissue adjacent to the tumor and from healthy tissue from the lobe without tumor were immediately frozen on liquid nitrogen, labeled and stored at −80°C for further analysis and the rest was perfused-fixed with 10% formaldehyde and 90% PBS at room temperature. Slides of liver tissue from normal and tumor growth were paraffin embedded and submitted for standard Hematoxylin and Eosin (H & E) staining. Blinded slides were assessed by two authors for evidence of tumor development. Tumors size and tumor volume were calculated from digital records. The largest tumor diameter and its calculated area were saved from each tumor using Digi3 Digital Binocular Microscope with DigiPro3.0 software (LaboMed, CA).

### 2.4. Labeling of Total Body Water and Blood Samples

F2 rabbits were injected intraperitoneally with saline made up in deuterated water 14 days prior and 10 days after the tumor implantation surgery. The volume of injection was calculated to achieve a 5% ^2^H-enrichment in body water, assuming that total body water accounts for 2/3 of body weight. Since the oxidation of foodstuffs and endogenous substrates yields unlabeled water, the rabbits were given 5% ^2^H-enriched drinking water for 72 hours. Blood draws (1.5 ml) were taken for 3 consecutive days from the marginal ear vein at days −13, −12, and −11 from tumor implantation (day 0) and at days +11, +12, and +13 after tumor implantation. The amount of blood and the number of draws were regulated by the Animal Committee at Case. All F2 animals were sacrificed after liver removal on day +14. Plasma was isolated after centrifugation at 3,000 rpm at 4°C for 10 min. To prevent its oxidation, GSH was immediately converted to a stable thioether by treating 100 *μ*l of plasma with 100 *μ*l of 50 mM iodoacetate in 10 mM ammonium bicarbonate, pH 10, adjusted with concentrated ammonia [18]. Aliquots were saved on liquid nitrogen and stored at −70°C for LC-MS/MS processing.

### 2.5. Mass Spectrometry Analysis

Glutathione species and ophthalmate concentrations and labeling were measured in plasma samples with mass spectrometry methods. Liquid chromatography-mass spectrometry (LC-MS/MS) processing details are described elsewhere [18]. *Homo*-glutathione was obtained from Chem-Impex International Inc. (Wood Dale, IL) and Ophthalmate from Bachem (Torrance, CA). Acetonitrile and DMSO were procured from Fisher Scientific (Pittsburgh, PA). In brief, a Hypersil Gold C18 column (2.1 × 150 mm, 5 *μ*m particle size; Thermo Electron Corp.) was kept at ambient temperature. Mobile phase A was 0.15% formic acid in water-acetonitrile (99 : 1, vol/vol), and mobile phase B was 0.15% formic acid in water-acetonitrile (5 : 95 vol/vol). Using a gradient elution, the compounds were eluted at a flow rate of 0.2 ml/min. The liquid chromatograph (Agilent 1100; Agilent Technologies Inc., Palo Alto, CA) was coupled to an API 4000 QTrap mass spectrometer (Applied Biosystems, Foster City, CA) operated under positive ionization mode. Analyst software (version 1.4.1; Applied Biosystems) was used for data registration. The MRM ion pairs were monitored to acquired data used to calculate isotopic enrichments from precursor→product pairs. The ion pairs monitored were (i) for carboxymethyl-GSH: 366.1→237.2, 367.2→237.1, and 367.2→238.1, (ii) for cyanomethyl-GSH derived from GSSR: 347.2→272.1, 348.2→218.1, and 348.2→219.1, (iii) for carboxymethyl-*homo*-glutathione: 380.1→233.1, (iv) for cyanomethyl-*homo*-glutathione: 361.1→232.1, and (v) for ophthalmate: 290.3 → 161.1, 291.3 → 161.1, 291.3 → 162.1. The M3 mol percent enrichment of the GSH derivative is calculated as M3/(M + M1 + M2 + M3). The [GSH]/[GSSG] ratio was calculated using the formula (GSH)/(GSSG/2). All samples were analyzed in triplicates.

Liver tissue was processed and submitted to Gas Chromatography-Mass Spectrometry (GC-MS) for measurement of cysteine. Powdered frozen tissue (25 mg) spiked with 5 nmol of *heptadecanoic acid* (C_17_) as internal standard was extracted with 2 ml of CH_3_CN/Methanol (1 : 1 precooled at −12°C and degassed with N_2_ flow) using a Polytron homogenizer. The slurry was centrifuged at 3800 rpm for 30 minutes at 4°C. The supernatant was collected and dried with Nitrogen gas flow. 

Derivatization was carried out in two steps: 30 *μ*l of 15 mg/ml of methoxyamine hydrochloride in dry pyridine was added to samples and incubated at 30°C for 90 minutes. After this 70 *μ*l of N-Methyl-N-trimethylsilyltrifluoroacetamide with 1% trimethylchlorosilane (MSTFA + 1% TMSC) was added and the mixture was incubated again at 37°C for 40 min. Analyses were carried out on an Agilent 5973 mass spectrometer, linked to a model 6890 gas chromatograph equipped with an autosampler. A Phenomenex ZB-5 MSi capillary column was used (30 m Length, 0.25 mm ID, 0.25 *μ*m film thickness). The carrier gas was helium (1.67 psig) and injection was 1 *μ*l in splitless mode. The GC temperature program was: initial temperature 60°C, hold for 1 min, increase by 10°C/min to 325°C, and hold 10 min. The injector temperature was set at 250°C and the transfer line at 275°C. EI source and quadrupole temperatures were set 250°C and 150°C, respectively. Raw data was deconvoluted with AMDIS software. Peak identification was carried out by matching retention time and mass spectra similarity against the Metabolomic Fiehn library (Agilent Technologies Inc, Santa Clara, CA). For further quantification, the data was exported to the SpectConnect server (Massachusetts Institute of Technology, Cambridge, MA). The relative concentration of cysteine was expressed as its relative area (divided by the area of the internal standard in the same chromatogram).

### 2.6. Statistical Analysis

Mean values ± standard deviations are presented (mean ± SD). Paired Students *t*-test was used to calculate significance of difference in the mean values from similar groups before and after tumor implantation (SPSS 16.0, Windows version licensed to Case Western Reserve University). ANOVA and one-sided independent Students *t*-test were used to calculate significance of difference in the mean values between the experimental group and the control group. A *P* value <.05 was considered statistically significant.

## 3. Results

**Figure 2:**
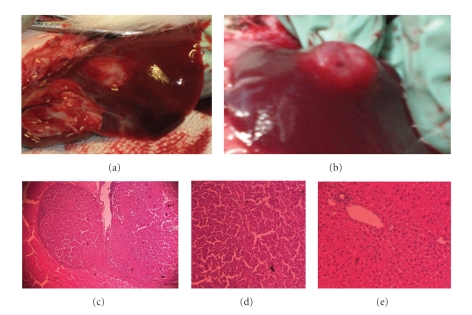
*Macroscopic and microscopic morphology of secondary liver tumors. *VX2 tumor cell grafts were implanted in liver from rabbits 14 days prior to tumor removal. All implanted tumor grafts showed macroscopic growth on liver sites. Some growths were observed to have a flat growth (a) while some others have a nodular growth (b). At histology, most tumors had a pseudocapsule formed by fibrotic tissue surrounded the tumor ((c) x20). Typical epithelial cells with malignant morphology are seen, however no neoangiogenesis, tumor invasion, or lymphocyte infiltration was seen at this stage of tumor growth ((d) x40). Typical normal liver rabbit tissue is seen in the rest of the organ ((e) x40).

**Figure 3:**
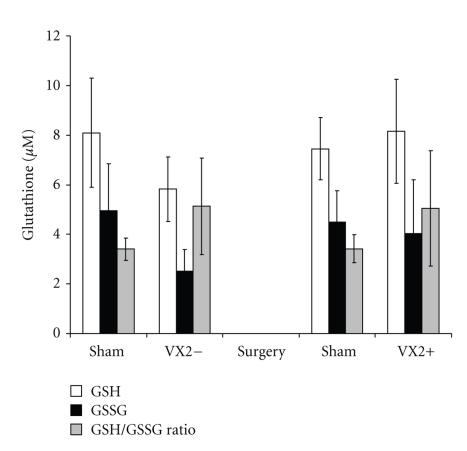
*Glutathione species in plasma from the VX2 rabbit model.* Both the reduced form (GSH) and the oxidized form (GSSG) of glutathione were measured in plasma from rabbits 2 weeks before and 2 weeks after tumor implantation. Levels of GSH, GSSG and GSH/GSSG ratio were similar from the experimental group and the sham group before and after tumor implantation (*P* > .05, paired *t*-test and one-sided independent *t*-test).

**Figure 4:**
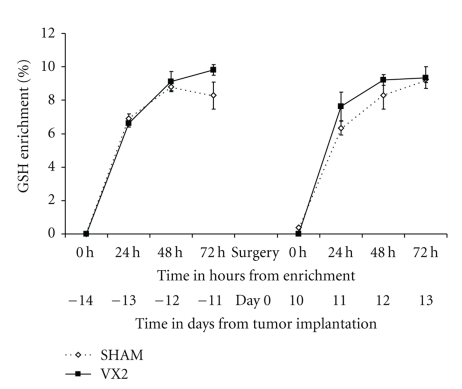
*Labeling of reduced glutathione (GSH) in plasma from the VX2 rabbit model. *The turnover of reduced glutathione (GSH) was measured in plasma from rabbits 2 weeks before and 2 weeks after tumor implantation. GSH was found to be similar in rabbits from the experimental group before and after tumor implantation and to sham animals (*P* > .05, paired *t*-test and one-sided independent *t*-test).

**Figure 5:**
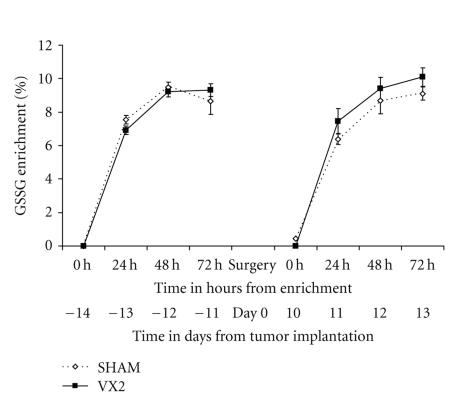
*Labeling of oxidized-bound glutathione (GSSG) in plasma from the VX2 rabbit model. *The turnover of oxidized-bound glutathione (GSSG) was measured in plasma from rabbits 2 weeks before and 2 weeks after tumor implantation. GSSR was found to be similar in rabbits from the experimental group before and after tumor implantation and to sham animals (*P* > .05, paired *t*-test and one-sided independent *t*-test).

Tumor growth was found in all six animals (100%) at the time of sacrifice two weeks after tumor implantation (Figure 2). The mean size of tumor growth (*n* = 12 tumors in 6 animals) was 8.4 ± 5.96 mm (mean ± SD of max. diameter) and tumor volume was 241.8 ± 78.6 mm^3^. Tumor volume was similar between lobes and among animals. Rabbits from the control (sham) group had normal livers with no visible Gelfoam on second laparotomy. Rabbits showed no sign of sickness prior to sacrifice or metastatic disease at sacrifice. Plasma concentrations of reduced glutathione (GSH) and oxidized-bound glutathione (GSSR) were not significantly different in rabbits from the experimental group before and after tumor implantation (*P* > .05, paired *t*-test) (Figure 3). Glutathione plasma concentrations from rabbits in the experimental group, either before or after tumor implantation, were similar to the ones in the sham group (*P* > .05, one sided *t*-test) (Figure 3). Furthermore, the labeling of GSH and GSSR in plasma was similar in experimental rabbits before and after tumor implantation (*P* > .05 by ANOVA and by independent student *t*-test at 24, 48, and 72 hours after deuterium enrichment) (Figures 4-5). Labeling of glutathione species in plasma, was also similar in experimental animals when compared to sham animals (*P* > .05).

**Figure 6:**
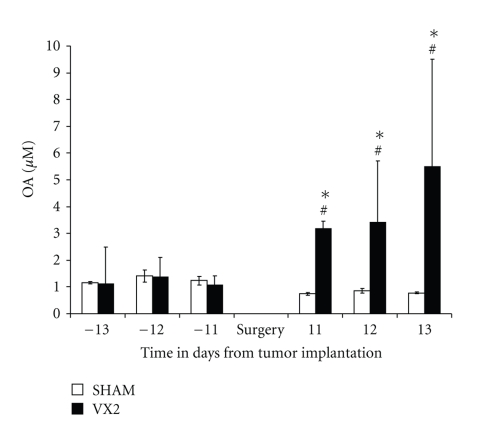
*Ophthalmate (OA) concentration in plasma from the VX2 rabbit model. *The concentration of OA was measured in plasma from rabbits 2 weeks before tumor implantation and 2 weeks after tumor implantation. Levels of OA were similar in rabbits from the experimental group before tumor implantation when compared to the control (sham) group (*P* > .05, one-sided independent *t*-test). In contrast, levels of OA were significantly higher in the experimental group when compared to the sham group after tumor implantation (*P* < .01, one-sided independent *t*-test). Furthermore, levels of OA were significantly higher in the experimental group in the state of tumor growth when compared to the state of health (*P* < .01, paired *t*-test).

**Figure 7:**
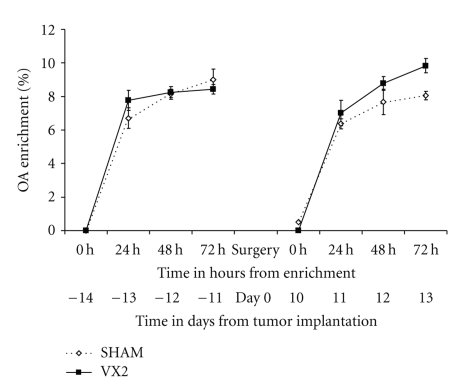
*Labeling of ophthalmate (OA) in plasma from the VX2 rabbit model. *The turnover of OA was measured in plasma from rabbits 2 weeks before tumor implantation and 2 weeks after tumor implantation. OA was found to be similar in rabbits from the experimental group before and after tumor implantation and to sham animals (*P* > .05, paired *t*-test and one-sided independent *t*-test).

**Figure 8:**
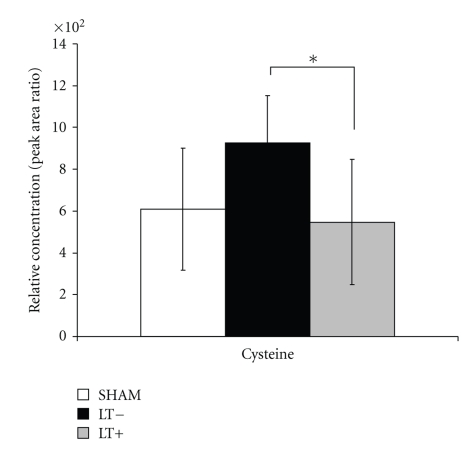
*Cysteine concentration in liver tissue from the VX2 rabbit model. *The concentration of cysteine was measured in liver tissue samples from experimental group (liver tissue adjacent to the tumor and liver tissue from the lobe without tumor) and liver tissue from sham animals. Levels of cysteine were found to be significantly lower in liver tissue adjacent to the tumor when compared to liver tissue from the lobe not containing tumor (*P* < .05 paired *t*-test). Relative concentrations of cysteine in liver tissue were similar between the experimental group (both liver adjacent to the tumor and liver away from tumor) when compared to the sham group (*P* > .05, independent *t*-test).

Ophthalmate plasma concentration before tumor implantation was similar in rabbits from the experimental group when compared to the control (sham) group (*P* > .05, one-sided independent *t*-test) (Figure 6). In contrast, plasma concentrations of ophthalmate were significantly higher in rabbits from the experimental group when compared to the sham group, after tumor implantation at all time points (*P* < .05, ANOVA and one-sided independent *t*-test). Furthermore, plasma concentrations of ophthalmate were significantly higher in rabbits from the experimental group in the state of tumor growth when compared to concentrations at their healthy state (*P* < .05, paired *t*-test). The plasma concentration of ophthalmate was similar in the control group before sham surgery when compared to the levels from the same group after sham surgery (*P* > .05, paired *t*-test). Plasma labeling of ophthalmate in experimental animals in their status of health was comparable to the labeling in their status of tumor growth and to sham animals (*P* > .05, by ANOVA and independent student *t*-test at 24, 48, and 72 hours after deuterium enrichment) (Figure 7). The relative concentration of cysteine, an amino acid precursor of GSH, was found to be significantly lower in liver tissue adjacent to the tumor when compared to liver tissue from the lobe not containing tumor (*P* < .05 paired *t*-test, Figure 8). Liver tissue relative concentrations of cysteine were similar between the experimental group (both liver adjacent to the tumor and liver away from tumor) when compared to the sham group (*P* > .05, independent *t*-test).

## 4. Discussion

We hypothesized that animals undergoing early tumor growth may be subject to an increase oxidative stress with disturbance of their GSH/GSSG redox buffer system, and a parallel increase in the production of ophthalmate. The present studies showed that both the concentrations as well as the labeling of glutathione species were similar in the state of health compared to the state of early tumor growth in the VX2 rabbit model. In contrast, the concentration of OA increased significantly from animals in the state of early tumor growth when compared to their healthy state and to sham animals. The small size of the ophthalmate pool makes OA a potential sensitive index of oxidative stress linked to variations in the rate of GSH synthesis and of cysteine availability [17]. The turnover of the glutathione species pool in the liver occurs rapidly, 2-3% per minute in the basal rate, even though its large size. While the plasma pool of GSH:GSSG is very small, its turnover is similar to that of the liver. Thus, the turnover of plasma GSH:GSSG is a proxy of the turnover of the liver GSH:GSSG [18]. In this animal model, a secondary liver tumor grew for two weeks compared to a control (sham) group. The concentrations of plasma GSH and GSSG as well as the [GSH]/[GSSG] ratio and their plasma labeling were similar in this model of early tumor growth compared to controls. Using the turnover of glutathione species in plasma as a proxy of the liver [18], the previous finding may suggest the strength of the glutathione redox buffer system in the nonaffected liver lobe in the early stage of tumor growth. Intracellular GSH oxidation in further developed to advanced and undifferentiated tumors is associated with an increase in glutathione peroxidase activity with further increase in its oxidized form GSSG and a significant decrease in the [GSH]/[GSSG] ratio in plasma [12–16].

The concentrations of plasma ophthalmate increased ~10-fold (*P* < .05) over the concentrations assayed in the same animals before tumor implantation and over the concentrations assayed in the control group before and after the sham operation. However, there was no difference in the profile of ^2^H-labeling of plasma ophthalmate. The first study to recognize ophthalmate as a biomarker for oxidative stress and hepatic GSH depletion [17] used a metabolomic approach to detect the changes that occur following acetaminophen-induced hepatotoxicity in mice; they found an ~5-fold increase in plasma and liver ophthalmate 1 hour after the administration of the drug, and this finding was associated with a depletion of GSH in liver tissue. No previous study to our knowledge has assessed oxidative stress metabolites as a possible diagnostic biomarker for liver cancer. Our results suggest that during early liver tumor growth there is a significant amount of oxidative stress resulting in increased intracellular glutathione oxidation, producing GSSG and depleting GSH in the affected part of the liver, suggested by the elevation of ophthalmate levels in plasma. Furthermore, analysis of liver tissue samples showed a decrease in the levels of cysteine, a precursor of GSH, in the immediate surroundings of the tumor when compared to the healthy side of the liver. Thus, we hypothesize that the GSH depletion associated to cysteine consumption described by Soga et al. in 2006 [17] is indeed a local phenomenon, in this case, associated with the early growth of a secondary liver tumor which may be causing oxidative stress in its immediate surroundings. While most of plasma GSH/GSSG comes from the liver, their total plasma concentrations and labeling were not affected in the experimental group. It may suggest the mass of liver tissue under stress was smaller in proportion to the mass of liver with a more normal metabolism. This ratio may change as the tumor grows.

Ophthalmate does not contain a thiol group, thus it does not participate in the many thiol-dependent reactions of glutathione. Since the proportion of ophthalmate to glutathione is small, the concentration of ophthalmate should be reflected more rapidly than that of plasma GSH:GSSG even though the turnover of ophthalmate remained constant. This makes ophthalmate a good model to study the disturbances of the glutathione buffer system during oxidative stress. The body redox systems, which include antioxidant enzymes and low molecular weight antioxidants, may be deregulated in cancer cells along with TNF-*α* and IL-6. Moreover, excessive concentrations of free radicals may overcome normal defense mechanisms and leave DNA at risk from oxidative modifications [5, 25]. This imbalance may enhance tumor growth and disease progression [26]. Higher rates of the oxidative stress metabolites appear to define the hallmark of early liver tumor growth which lends to the potential application for ophthalmate to be used as a surrogate for early liver growth. The role of ophthalmate in a cirrhotic liver or in primary liver tumors remains to be determined in further studies.

The findings of our study must be interpreted in light of some limitations. Increment of ophthalmate concentration was observed in a very early stage of secondary tumor growth. Due to the nature of the animal model chosen, we do not know if these findings will be similar in subsequent stages of tumor growth, that is, tumor invasion, spread or in cases of primary liver tumors. Concomitant conditions such as hepatitis virus infection or portal hypertension may confound these findings. We made an attempt to correlate the levels of glutathione species, and ophthalmate with the tumor mass; however, due to the design of the study there were no significant differences in tumor size/volume in animals from the experimental group. In addition, the oxidative stress status of an animal with a tumor growth may be a function not only of the tumor nature and its mass, but among other factors, their cytokine milieu, and nutritional status. In spite of these boundaries, the present studies showed that metabolic disturbances in animals with early liver tumor growth can be detected and, perhaps metabolic signatures of liver tumor growth, may overcome the limitations of current biomarkers used in the clinical setting. Currently, there are many liver biomarkers under investigation. A few of them recently reported include des-gamma carboxyprothrombin, lens culinaris agglutinin-reactive *α*-fetoprotein, human hepatocyte growth factor, and insulin-like growth factor-1 [27]. Their clinical relevance is still under study.

In conclusion, the plasma concentration of ophthalmate significantly increased in rabbits after early liver tumor implantation and growth. This finding reflects an increase in the oxidative stress placed on the local liver tissue during early tumor growth. Ophthalmate may prove to be a surrogate of tumor subsistence and early tumor growth.

## Abbreviations

OA: Ophthalmate
GSH: Glutathione reduced
GSSG: Glutathione oxidized
^2^H_2_O: Deuterated water
TBW: Total body water
HCC: Hepatocellular carcinoma
AFP: *α*-fetoprotein
CEA: Carcinoembryonic antigen
TNF-*α*: Tumor necrosis factor-*α*
OS: Oxidative stress
GPx: Glutathione peroxidase
IP: intraperitoneal
IM: intramuscular
SC: subcutaneous
FBS: Fetal bovine serum
DMEM: Dulbecco's modified eagle medium
HBSS: Hank's balanced salt solution
LC-MS: Liquid chromatography-mass spectrometry
GS-MS: Gas chromatography-mass spectrometry.
